# Mdm2 inhibition confers protection of p53-proficient cells from the cytotoxic effects of Wee1 inhibitors

**DOI:** 10.18632/oncotarget.5891

**Published:** 2015-09-29

**Authors:** Yizhu Li, Priyanka Saini, Anusha Sriraman, Matthias Dobbelstein

**Affiliations:** ^1^ Institute of Molecular Oncology, Göttingen Centre of Molecular Biosciences (GZMB), Faculty of Medicine, University of Göttingen, Göttingen, Germany

**Keywords:** Wee1, Mdm2, p53, gemcitabine, premature mitosis

## Abstract

Pharmacological inhibition of the cell cycle regulatory kinase Wee1 represents a promising strategy to eliminate cancer cells. Wee1 inhibitors cooperate with chemotherapeutics, e. g. nucleoside analogues, pushing malignant cells from S phase towards premature mitosis and death. However, considerable toxicities are observed in preclinical and clinical trials. A high proportion of tumor cells can be distinguished from all other cells of a patient's body by inactivating mutations in the tumor suppressor p53. Here we set out to develop an approach for the selective protection of p53-proficient cells against the cytotoxic effects of Wee1 inhibitors. We pretreated such cells with Nutlin-3a, a prototype inhibitor of the p53-antagonist Mdm2. The resulting transient cell cycle arrest effectively increased the survival of cells that were subsequently treated with combinations of the Wee1 inhibitor MK-1775 and/or the nucleoside analogue gemcitabine. In this constellation, Nutlin-3a reduced caspase activation and diminished the phosphorylation of Histone 2AX, an indicator of the DNA damage response. Both effects were strictly dependent on the presence of p53. Moreover, Nutlin pre-treatment reduced the fraction of cells that were undergoing premature mitosis in response to Wee1 inhibition. We conclude that the pre-activation of p53 through Mdm2 antagonists serves as a viable option to selectively protect p53-proficient cells against the cytotoxic effects of Wee1 inhibitors, especially when combined with a nucleoside analogue. Thus, Mdm2 antagonists might prove useful to avoid unwanted side effects of Wee1 inhibitors. On the other hand, when a tumor contains wild type p53, care should be taken not to induce its activity before applying Wee1 inhibitors.

## INTRODUCTION

Inhibitors of the kinase Wee1 are capable of inducing cancer cell death with high efficiency, in particular when combined with chemotherapeutics such as nucleoside analogues [1] or platinum compounds [2]. In particular, the Wee1 inhibitor MK-1775 has been found efficient to eliminate a number of cancer cell species [3, 4], and it is currently evaluated in numerous clinical trials ([5] and 21 entries to clinicaltrial.gov).

Wee1 is a cell cycle regulatory kinase. It phosphorylates and thereby inactivates the downstream cyclin dependent kinases (CDKs) 1 and 2 [6, 7]while the cell replicates its DNA (i. e. in S phase) [8]. This suppression of CDKs ensures that the cell will first complete the replication of the entire genome before moving on to mitosis. Removing Wee1 by siRNA, or inhibiting Wee1 by small compounds, results in the premature onset of mitosis, thereby increasing cell death [3, 4].

Wee1 inhibition can be regarded as a way to exploit replicative stress for cancer treatment, as we have reviewed recently [9]. Tumor cells often display impaired abilities to ensure a smooth and uninterrupted replication of their DNA. Further increasing this stress situation represents a viable strategy of cancer therapy. This can be achieved by classical chemotherapeutics, e. g. nucleoside analogues. Representatives of this class include gemcitabine (2′, 2′-difluorodeoxycytidine, dFdC), an analogue of deoxycytidine. Gemcitabine inhibits ribonucleotide reductase, thus leading to a shortage and imbalance of available deoxyribonucleotide triphosphates. Moreover, it is incorporated into newly synthesized DNA, leading to torsional stress and replication fork stalling [10]. Interfering with the replication machinery is one example of targeting tumor-supportive cellular machineries for cancer treatment, as reviewed recently [11].

Wee1 inhibition and the consecutive activation of CDK1 can exacerbate replicative stress by at least three mechanisms. Firstly, we have recently identified a mechanism that leads from Wee1 inhibition to the inactivation of Chk1, a key enzyme required to re-enable DNA replication in the context of replicative stress [12]. Moreover, Wee1 inhibition increases nucleotide consumption and thereby increases replicative stress [13]. On top of this, however, Wee1 inhibition, by enabling premature CDK activity during S phase, promotes mitosis despite the fact that their DNA is incompletely replicated [1]. This will either disable the completion of mitosis, resulting in catastrophic death, or otherwise lead to the formation of two daughter cells with gross genetic deletions, again precluding survival.

Despite the encouraging preclinical and clinical findings, Wee1 inhibitors have not achieved clinical approval yet. One of the problems faced when evaluating these drug candidates consisted in the toxicity that limited the amount of inhibitors that can be safely administered. Such dose limiting toxicities include myelosuppression and tachyarrhythmia [5]. In other words, a better distinction between normal cells and the tumor cells in a patient's body is required, and the cytotoxic effects should be limited to the tumor cells as much as possible.

The most frequent genetic difference between tumor cells and normal cells consists in mutations within the gene *TP53*, encoding the tumor suppressor and transcription factor p53 [14]. When activated, e. g. by phosphorylation through DNA damage-induced kinases, p53 induces the expression of genes that induce cell cycle arrest in G1 or G2. Strong p53 activation, e. g. by excessive DNA damage, can also induce cell death, most notably by apoptosis [15]. More than 50% of all tumors, however, carry an inactivating mutation in TP53. This typically disables the encoded p53 protein from binding to its cognate promoter sequences, precluding transactivation. In these cases, pharmacological activation of p53 will only pertain to normal cells but not to tumor cells.

p53 activity is kept under tight control by its antagonist Mdm2. Mdm2 binds and inactivates p53, and on top of this, it acts as an E3 ubiquitin ligase to target p53 for proteasomal degradation. The synthesis of Mdm2 is induced by p53, leading to a negative regulatory feedback loop. Small molecule inhibitors have been developed to bind Mdm2, precluding p53 from binding to the same site. As a result, these drugs can be used to augment the levels of active p53, even in the absence of DNA damage [16]. The prototype compound of this kind has been termed Nutlin-3a [17], but several similar drug candidates have been developed since and are currently under evaluation in clinical studies [18].

While mostly regarded as an inhibitor of cell survival, p53 can also be employed to protect cells. To this end, Mdm2 inhibitors can be employed to activate p53. We have first described the protective effect of Mdm2 inhibition in the case of nucleoside analogues, e. g. gemcitabine [19]. Since p53 arrests cells in G1 or G2, few cells replicate their DNA upon p53 activation by Mdm2 inhibitors, and nucleoside analogues can no longer be incorporated into nascent DNA strands. As a result, the cells become resistant to treatment with nucleoside analogues. When both drugs are washed off, the cells can resume proliferation with only short delays. An analogous approach was used to achieve protection against taxanes, i. e. drugs that target the mitotic spindle. Pre-treatment with Nutlin-3a precludes cells from entering mitosis, the most vulnerable phase of cells in the face of taxanes, and it thus ensures cell survival [20]. The protective effect of Mdm2 against mitotic inhibitors is active for several days and can be further enhanced by rapamycin [21]. Thus, p53 activation can provide protection of p53-proficient cells against specific classes of drugs. This strategy dates back to the beginning of the millennium, when low-dose DNA-damaging agents provided protection against microtubule-active drugs through p53 [22, 23], a principle termed cyclotherapy [24].

Here we show that Mdm2 inactivation successfully protects p53-proficient cells against the cytotoxic effects of Wee1 inhibition. When p53 is pre-activated, Wee1 inhibitors alone or in combination with gemcitabine no longer prevent long term proliferation and survival. Mechanistically, p53-activation keeps cells from the lethal premature mitosis that is otherwise induced by Wee1 inhibition.

## RESULTS

### Mdm2 inhibition allows cells to survive the treatment with Wee1 inhibitor and/or gemcitabine

**Figure 1:**
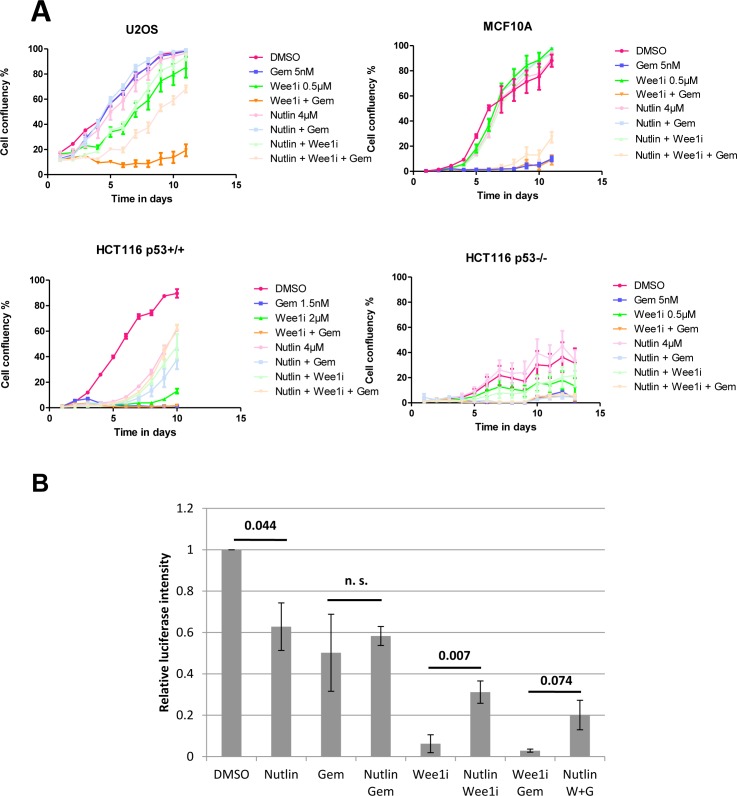
Nutlin protects cells against Wee1 inhibition and/or gemcitabine **A.** U2OS, MCF10A, HCT116 p53+/+, and HCT116 p53−/− cells were treated with Nutlin-3a for 24 hrs, followed by treatment with MK-1775 (Wee1i), gemcitabine and continuous incubation with Nutlin, at the indicated drug concentrations. After another 24 hrs, all drugs were removed and fresh medium was added. Cells were incubated for 8-13 days and confluency was measured each day using brightfield microscopy (Celigo cell cytometer). Error bars represent the SD, n=3 (triplicate experiments). **B**. U2OS cells were treated with 8μM Nutlin for 24 hrs, followed by treatment with 1μM Wee1i and 300nM gemcitabine, along with continuous treatment with 8μM Nutlin. At 72 hrs, the cells were lysed using the CellTiter-Glo®Reagent, and cell viability was measured via an ATP-dependent luciferase signal. Student's T-test p-values are stated above the horizontal bars. Error bars represent the SE, n=3.

To assess whether pre-treatment with an Mdm2 inhibitor affects the survival of p53-proficient cells, we first treated U2OS cells (human osteosarcoma, p53 wild type) with Nutlin-3a, the prototype pharmacological antagonist that binds to Mdm2 and precludes its interaction with p53 [17]. After a 24 hrs incubation time, the cells were treated with gemcitabine and/or the Wee1 inhibitor MK-1775 (termed Wee1i from here on) for another 24 hrs, while maintaining the concentration of Nutlin-3a (simply termed Nutlin from here on) as before. For each drug, control experiments using the DMSO solvent were performed in parallel. Subsequently, all drugs were washed off, followed by further incubation in regular cell culture media. For twelve days, the cell density was monitored by transmission light microscopy and automated image analysis (Fig. 1A). Gemcitabine alone did not lead to a strong impairment of cell proliferation, and also Wee1i alone only moderately prevented cell growth. When applied together at the same concentrations, however, the two drugs strongly reduced the appearance of proliferating cells, essentially preventing the formation of a confluent layer, confirming the synergy that was described before [12, 25-28]. Strikingly, the pre-treatment with Nutlin rescued the proliferation of cells that were treated with Wee1i alone, and even more strongly reversed the effect of Wee1i and gemcitabine in combination. Parallel experiments were performed with the non-transformed cell line MCF10A. Interestingly this cell line was largely resistant to Wee1 inhibition. However, the cells responded to Gemcitabine or the combination of Gemcitabine with Wee1i, and in both cases, this effect was alleviated by Nutlin. To define the role of p53 in the protection by Nutlin, we employed HCT116 cells, a colon cancer-derived cell line that had been engineered to either contain or lack wild type p53 [36]. In the case of HCT116 p53+/+ cells, we observed that cell proliferation on treatment with gemcitabine or Wee1i, and also upon co-treatment with Wee1i, was strongly reduced. However, in combination with Nutlin, we observed a rescue in cellular proliferation. In HCT116 p53−/− cells, however, no such rescue by Nutlin was observed. Thus, the protective effect of Nutlin is p53-dependent. We conclude that pre-treatment with Nutlin has an intense protective effect and allows cells to survive the treatment with Wee1i, alone or in combination with gemcitabine.

Next, we investigated whether Nutlin pre-treatment also affects immediate cell viability when cells are exposed to gemcitabine and/or Wee1i. To test this, we treated U2OS cells as above, followed by a 72 hrs incubation and a viability assay based on the determination of cellular ATP levels by luciferase (Fig. 1B). All three drugs – gemcitabine, Nutlin, and Wee1i – led to a reduction in viability, presumably through a combination of cell death and arrested proliferation. Wee1i, alone or in combination with gemcitabine, reduced viability most strongly. Importantly, however, Nutlin rescued the viability of Wee1i-treated cells, with or without gemcitabine. Thus, Nutlin pre-treatment strongly protects cells from the induction of death by Wee1i.

### Mdm2 inhibition attenuates caspase activity and the phosphorylation of Histone2AX in response to Wee1 inhibition

**Figure 2:**
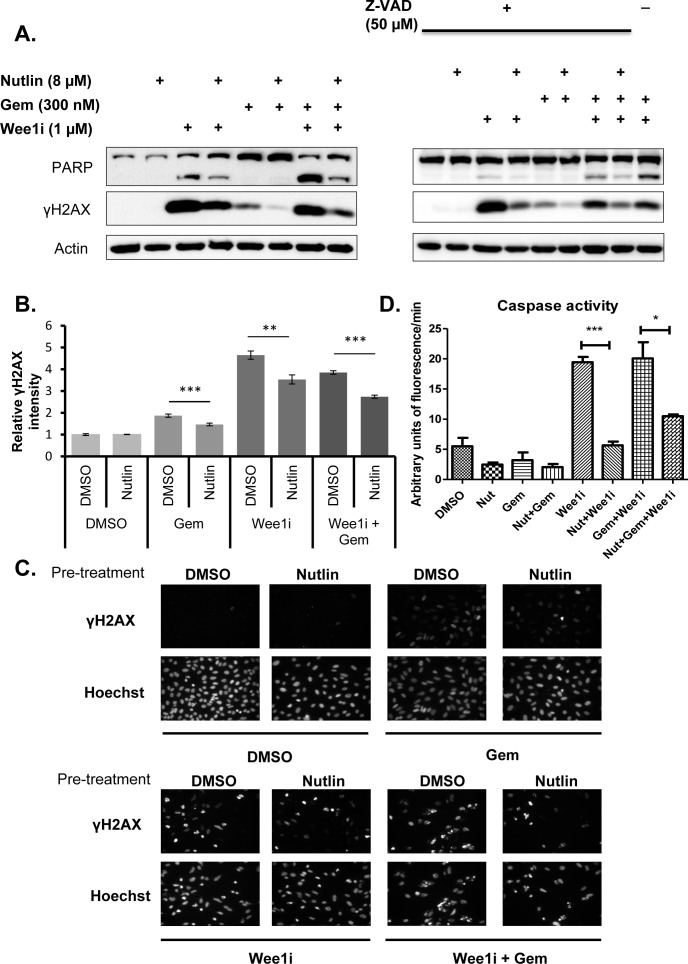
Nutlin prevents caspase activation and γH2AX accumulation in response to Wee1 inhibitor and/or gemcitabine **A**. U2OS cells were treated with 8μM Nutlin for 24 hrs, followed by treatment with 1μM Wee1 inhibitor, 300nM gemcitabine, and/or 8μM Nutlin in the absence and presence of 50μM ZVAD-FMK for another 24 hrs. Cells were harvested and immunoblot analysis was performed to detect poly-ADP ribose polymerase (PARP) and γH2AX. **B**., **C**. U2OS cells were treated as in (A). The cells were then fixed and stained for γH2AX by immunofluorescence. Detection and analysis was performed using automated immunofluorescence microscopy (BD Pathway). Figure panel (B) shows images of γH2AX staining for each treatment condition. Quantitation of γH2AX intensities was done using the BD pathway analysis tool and depicted in figure panel (C). Error bars represent the SD, n=3. **D**. U2OS cells were treated with 8μM Nutlin for 24 hrs, followed by treatment with 1μM Wee1 inhibitor, 300nM gemcitabine, 8μM Nutlin in the absence and presence (Supplementary Figure 1) of 50μM ZVAD-FMK for another 24 hrs. The cells were harvested and lysed for caspase activity assay. Fluorescent intensity measurements were obtained for each treatment. The activity (arbitrary units of fluorescence/min) was calculated for each treatment at the linear part of the curve (cf. Supplementary Figure 1). Error bars represent the S.D, n=3.

Wee1i exerts its toxic effects, at least in part, by inducing a DNA damage response [8, 13] and apoptosis [29]. We therefore tested whether Nutlin pre-treatment reduces any or both of these responses. U2OS cells were pre-treated with Nutlin or the DMSO solvent, followed by gemcitabine and/or Wee1i. Subsequently, the cleavage of poly ADP-ribose polymerase (PARP), a bona fide caspase substrate [30], was monitored by immunoblot analysis; we also probed the phosphorylation of Histone2AX (γH2AX), a hallmark of the DNA damage response [31] (Fig. 2A). Wee1i induced PARP cleavage as well as a strong accumulation of γH2AX in the presence or absence of gemcitabine, as reported previously [12]. Notably, however, both responses were clearly reduced when the cells had been pre-treated with Nutlin. Similar results were obtained when blocking caspase activities by the cell-permeant pan caspase inhibitor Z-VAD-FMK, suggesting that γH2AX levels represent the direct result of a DNA damage response, not an indirect consequence of caspase activation. To confirm the reduction in γH2AX independently, we assessed its accumulation by immunofluorescence and subsequent digital image analysis (Fig. 2B and 2C), as described [32]. We observed the accumulation of γH2AX upon treatment with gemcitabine and Wee1i, alone or in combination. In each case, however, Nutlin pre-treatment led to a highly significant reduction in the accumulation of γH2AX. Finally, we assessed the activity of caspases in cell lysates obtained from U2OS cells after drug treatment. We observed increased activities in samples treated with Wee1i, alone or and in combination with Gemcitabine; again, however, this was rescued upon pre-treatment with Nutlin (Fig. 2D; Suppl. Fig. 1). In control samples treated with Z-VAD-FMK, no caspase activity was observed, validating the assay. Taken together, Mdm2 inhibition attenuates both the activation of caspases as well as DNA response signaling upon inhibition of Wee1.

### The presence of p53 is required for the protective effect of Nutlin-3a against Wee1 inhibition

**Figure 3:**
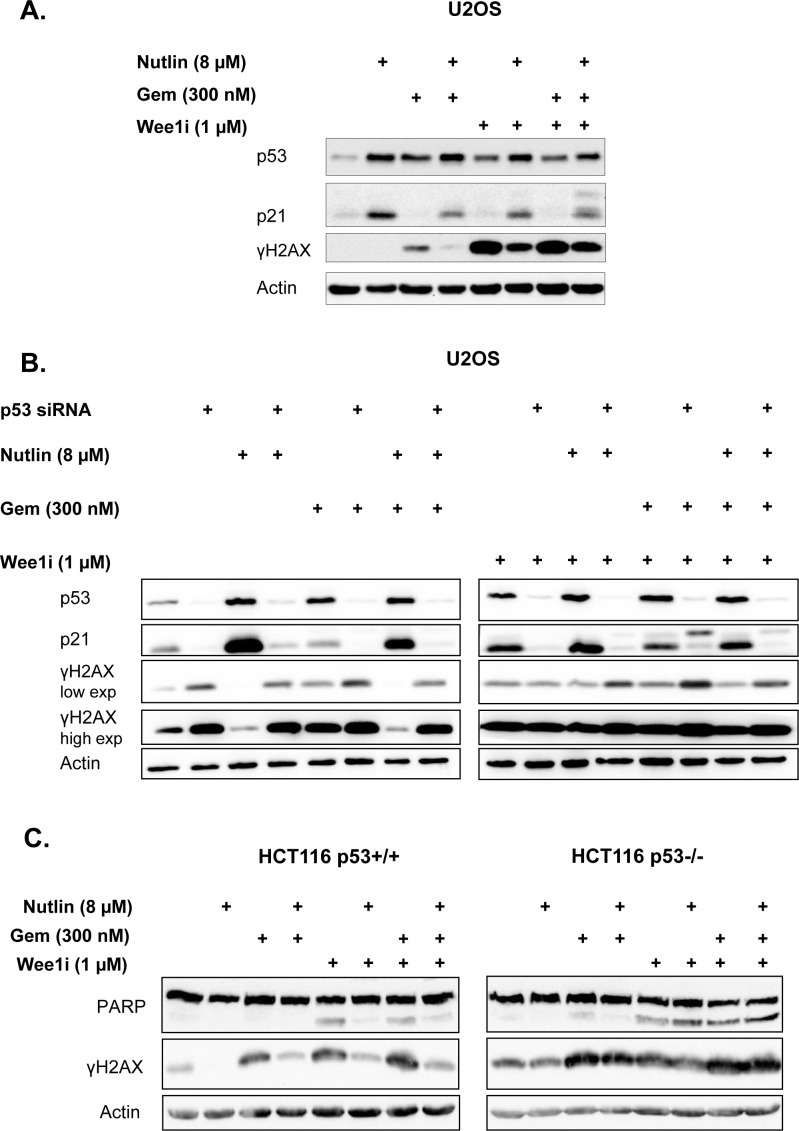
p53 is required for the protective effect of Nutlin **A**. U2OS cells were treated with 8μM Nutlin for 24 hrs, followed by treatment with 1μM Wee1 inhibitor, 300nM gemcitabine and 8μM Nutlin for another 24 hrs as indicated. Cells were harvested and immunoblot analysis was performed to detect p53 and its target gene product p21. **B**. U2OS cells transfected with siRNA were treated with 8μM Nutlin at 24 hrs post-transfection, then incubated for additional 24 hrs, followed by treatment with 1μM Wee1 inhibitor, 300nM gemcitabine and 8μM Nutlin for another 24 hrs as indicated. Immunoblot analysis was performed to detect p53 and its target gene product p21, as well as γH2AX. **C**. An isogenic pair of HCT116 cells with or without a targeted deletion of *TP53* was pre-treated with 8μM Nutlin for 24 hrs, followed by treatment with Wee1 inhibitor, gemcitabine and Nutlin for another 24 hrs. Cells were harvested and subjected to immunoblot analysis to detect PARP and γH2AX.

Mdm2 is mostly known for its impact on p53, but additional activities of Mdm2 have been reported [33], and some of them may be affected by Mdm2 antagonists as well. To assess whether Nutlin antagonizes Wee1i through p53, we first assessed whether it increases the levels of p53 and the product of a target gene, CDKN1A/p21 [34], in U2OS cells (Fig. 3A). As expected, Nutlin led to the accumulation of p53 as well as p21. Importantly, neither the subsequent treatment with gemcitabine nor the exposure to Wee1i led to any gross changes in the levels of p53 or p21 when cells had been pre-treated with Nutlin. We did, however, observe the accumulation of p53 but not p21 when the cells were treated with gemcitabine and/or Wee1i alone. This is in agreement with previous analyses indicating that DNA damage (as observed by γH2AX accumulation) during S phase stabilizes p53 but nonetheless attenuates the induction of p21 [35]. In any case, the effects of Nutlin on p53 levels and activity were not compromised by gemcitabine and/or Wee1i.

To define the role of p53 in the negative impact of Nutlin on γH2AX accumulation, we transfected U2OS cells with siRNA to p53. This knockdown abolished the influence of Nutlin on γH2AX (Fig. 3B), indicating that the protective effective of Nutlin against Wee1i depends on p53.

Next, we assessed the protective effect of Nutlin in a system of isogenic cells that only differ in their p53 status. HCT116 cells that either contained or lacked wild type p53 [36] were employed for this purpose. Again, these cells were pre-treated with Nutlin, followed by gemcitabine and/or Wee1i, and the accumulation of cleaved PARP as well as γH2AX was assessed by immunoblot analysis (Fig. 3C). In the case of cells containing wild type p53, Nutlin prevented both caspase activity and the DNA damage response, similar to U2OS cells. When *TP53* had been deleted, however, Nutlin did not influence any of these responses. In conclusion, p53 is strictly required for the protective effects of Nutlin against Wee1i. Thus, p53 activity is the principal mediator of this protection.

### Nutlin-3a prevents the accumulation of cells in premature mitosis when exposed to Wee1 inhibitor

**Figure 4:**
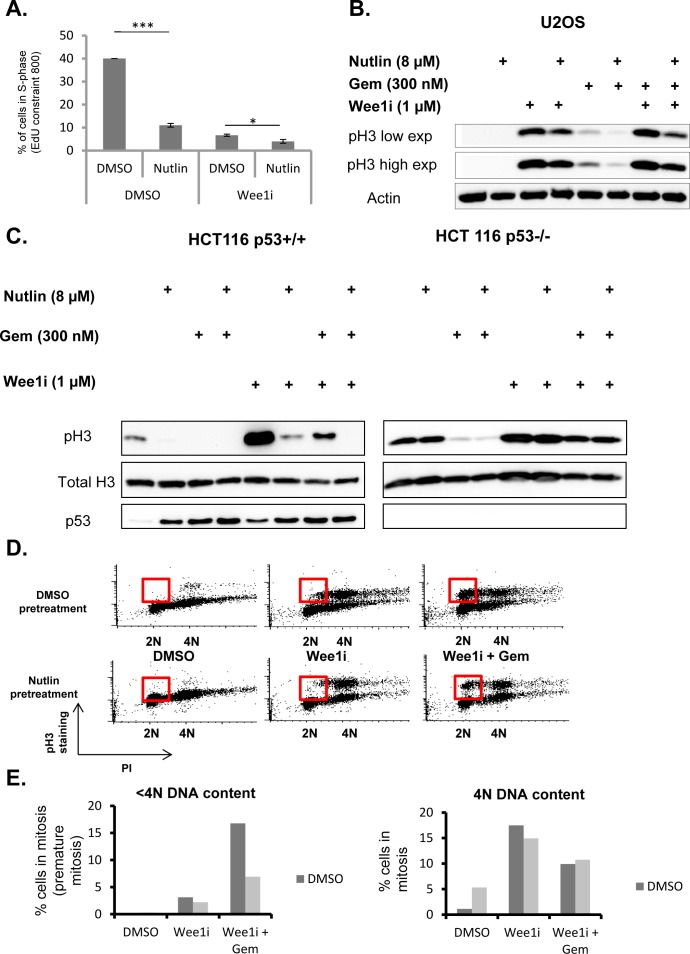
p53 prevents accumulation of cells in premature mitosis A. U2OS cells were treated with 8μM Nutlin for 24 hrs, followed by treatment with 1μM Wee1 inhibitor and 8μM Nutlin for another 24 hrs. Two hours before fixation, 5μM of 5-Ethynyl-2′-deoxyuridine (EdU) was added. Afterwards, cells were stained for EdU, and the percentage of cells with EdU staining intensities of 800 unitsor more was plotted. Error bars represent the SD, n=3. **B**. U2OS cells were treated with 8μM Nutlin for 24 hrs, followed by treatment with 1μM Wee1 inhibitor, 300nM gemcitabine and 8μM Nutlin for another 24 hrs. Immunoblot analysis was performed to detect Histone3 with a phosphorylation at Serine 10, a hallmark of mitosis. **C**. An isogenic pair of HCT116 cells with or without a targeted deletion of *TP53* was pre-treated with 8μM Nutlin-3 for 24 hrs, followed by treatment with Wee1 inhibitor, gemcitabine and Nutlin for another 24 hrs. Cells were harvested and subjected to immunoblot analysis to detect Histone H3 with a phosphorylation at Serine 10, p53, and total histone H3. **D**., **E**. U2OS cells were pre-treated with nutlin-3 as in Fig. 2B and 2C, followed by treatment with 1μM Wee1 inhibitor, 300nM gemcitabine and 8μM Nutlin for 8 hrs. The cells were fixed, stained for phospho-H3 along with propidiumiodide (PI) labelling, and analyzed by flow cytometry. The red boxes demarcate cells in premature mitosis. Figure panel (D) represents the percentage of cells stained positive for phospho-H3.

Wee1 acts to prevent the premature onset of mitosis, and its inhibition is known to trigger chromosome condensation and cell division, even before the replication of cellular DNA is complete. This condition – often referred to as premature mitosis – leads to a catastrophic situation and cell death [1]. Premature mitosis is even further enhanced when Wee1 inhibitors are combined with DNA-damaging agents, such as nucleoside analogues or platinum compounds [12, 25, 26, 28]. On the other hand, p53 often prevents even the entry of cells into S phase, or otherwise acts to block the transition into mitosis [37]. We therefore tested whether Mdm2 inhibition and p53 activation might prevent premature mitosis when cells are exposed to Wee1i. Firstly, we determined the amount of U2OS cells actively synthesizing DNA upon pre-treatment with Nutlin and/or subsequent treatment with Wee1i (Fig. 4A). Nutlin strongly reduced the number of cells in S phase, as determined by the incorporation of the labeling nucleoside analogue 5-ethynyl-2′-deoxyuridine (EdU) andreported previously [19]. Notably, the treatment with Wee1i also reduced the amount of EdU-incorporating cells, presumably due to interruptions in S phase. However, even in this situation, Nutlin further reduced the percentage of DNA-synthesizing cells, arguing that Nutlin keeps cells out of S phase regardless of subsequent Wee1i treatment. And indeed, propidium iodide staining of the cells revealed that Nutlinpretreated cells were largely accumulating with a DNA content corresponding to G1 or G2/M, regardless of their subsequent treatment (Suppl. Fig. 2).

Next, we compared the extent of entry into mitosis when U2OS cells were treated with Wee1i and/or gemcitabine, in the presence or absence of Nutlin pre-treatment. Wee1i, alone or in combination with gemcitabine, strongly augmented the accumulation of Histone3 (H3) that was phosphorylated at Serine 10, a marker of cells in mitosis [38] (Fig. 4B). Of note, however, Nutlin pre-treatment reduced the phospho-H3 signal in all combinations of Wee1i and gemcitabine. Thus, Nutlinpre-treatment reduces the accumulation of mitotic cells upon exposure to Wee1i.

Similar investigations were carried out in isogenic HCT116 cells with or without p53. Again, these cells were pre-treated with Nutlin, followed by gemcitabine and/or Wee1i, and the accumulation of Histone 3 (H3) that was phosphorylated at Serine 10 was assessed by immunoblot analysis (Fig. 4C). As expected, Wee1i increased the levels of phospho-H3, whereas Nutlin prevented this accumulation. Importantly, however, this was only observed in p53-proficient cells. When p53 was absent, Wee1 inhibition still induced phospho-H3 accumulation, but this was not affected by Nutlin.

Immunoblot analysis does not distinguish between regular and premature mitosis. In order to find out how Nutlin affects the accumulation of cells that prematurely enter cell division, we treated U2OS cells with combinations of the three drugs, followed by two-dimensional flow cytometry, quantifying both the DNA content and the amount of phosphorylated H3 in every cell (Fig. 4D and 4E). Cells with a DNA content below 4N but a phospho-H3 content above the baseline were considered prematurely mitotic. As expected, Wee1i led to the accumulation of cells in premature mitosis, especially when combined with gemcitabine. However, this number was strongly reduced when pre-treating the cells with Nutlin. We conclude that Nutlin prevents premature mitosis in cells that are confronted with Wee1i, alone or in combination with gemcitabine. We propose that this mechanism is at least partially responsible for the protection of Wee1i-treated cells against Nutlin.

## DISCUSSION

**Figure 5:**
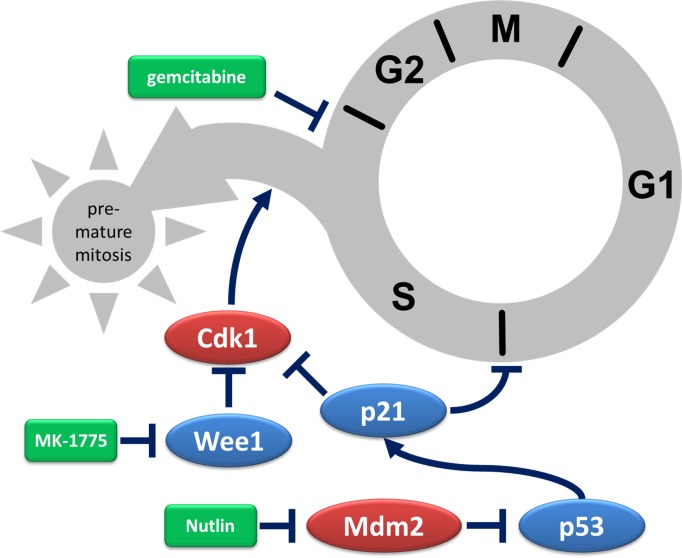
Depiction of protective mechanisms triggered by Mdm2 inhibition Gemcitabine halts progression through S phase by interfering with DNA replication. In the presence of Wee1 inhibitors, hyperactive CDK1 triggers premature mitosis despite incomplete DNA replication, usually resulting in cell death. When Mdm2 inhibitors activate p53 and thereby increase the levels of the CDK inhibitor p21, the transition from G1 to S phase is inhibited. Moreover, CDK1 inhibition by p21 reduces premature entry in mitosis. Taken together, pre-treatment of p53-proficient cells with an inhibitor of Mdm2 attenuates the cytotoxic effects of Wee1 inhibition. In the scheme, activators of cell cycle progression are depicted in red, inhibitors of cell cycle progression in blue, and drugs in green. Arrows indicate activation, lines that end with a bar indicate inhibition.

According to our results, the pharmacological inhibition of Mdm2 prevents the toxicity of a Wee1 inhibitor, in the presence or absence of the nucleoside analogue gemcitabine. In agreement, the Mdm2-inhibitor Nutlin prevents the accumulation of phosphorylated H2AX and the activation of apoptosis in response to Wee1i. As expected, this protective effect conferred by Nutlin strictly requires the presence of p53. Mechanistically, p53 diminishes the onset of premature mitosis by Wee1i and/or gemcitabine. We propose that Nutlin, by inducing the CDK inhibitor p21, interferes with G1-S transition and thus prevents replicative stress in the first place. In addition, p21 also attenuates CDK1 activity [39] and may thereby diminish premature mitosis even in those cells that nonetheless entered S phase (Fig. 5).

These observations are suggesting two major conclusions. Firstly, the therapeutic effect of Wee1 inhibitors may be reduced or even abolished if wild type p53 is activated prior to Wee1 inhibition. This not only argues against the combination of Mdm2 inhibitors with Wee1 inhibitors to treat p53-wildtype cancers. Rather, p53 is activated by most DNA-damaging therapeutic regimens, including irradiation and chemotherapy, e. g. by platinum compounds, topoisomerase inhibitors, alkylating agents, and many others [40]. When combining any of these chemotherapeutics with Wee1 inhibitors, it appears advisable to administer the Wee1 inhibitor before or at least simultaneously with chemotherapy, but not shortly after it. Otherwise, it is conceivable that the pre-activated p53 will interfere with cell cycle progression and thus with the efficacy of Wee1 inhibitors. In contrast, the presence of wild type but not pre-activated p53 does not seem to preclude the cytotoxic activity of a Wee1 inhibitor [41]. Notably, these considerations only applies to tumors that retain wild type p53, thus in about 50% of all human malignancies.

Secondly, our results suggest a strategy that may ultimately help to prevent unwanted toxicities of Wee1 inhibitors. Such toxicities, e. g. myelosuppression and tachyarrhythmia, have been reported [5] and may currently limit the usefulness of this class of drugs, especially when combining them with conventional chemotherapy, and despite their highly promising anti-cancer activity in preclinical models [2, 25, 26, 28, 42-47]. In those cases where p53 is absent or mutant and thus unable to activate its target genes, the administration of Nutlin or similar Mdm2 inhibitors will not interfere with the efficacy of Wee1 inhibitors against tumor cells, as exemplified by p53−/− HCT116 cells in this study (Fig. 3B). However, normal cells from such patients still contain wild type p53. The reversible activation of p53 by an Mdm2 antagonist can thus be expected to attenuate the toxic effects imposed by Wee1 inhibitors on non-cancerous cells. Thus, besides their use to eliminate cancer cells that contain wild type p53 but hyperactive Mdm2, Mdm2-inhibitors may prove useful as a means to prevent unwanted side effects of Wee1 inhibitors.

Such a use of Mdm2 inhibitors for avoiding the toxicities of cancer treatment is not limited to Wee1-inhibitors. Rather, Nutlin and related compounds were suggested to prevent the side effects of other anti-cancer compounds. We have previously found that Nutlin also acts to reduce the toxicities of nucleoside analogues in p53-proficient cells [19], and similar protective effects have been reported for taxanes[20, 48], HDAC inhibitors [49], resveratrol, [50], and other chemotherapeutics [51-56]. Furthermore, the protective effect of Mdm2-inhibitors is to be expected for any compound that affects cell survival predominantly in S or M phase. This includes inhibitors of Chk1 [57] and ATR [58], which increase replicative stress and promote premature mitosis; when combined with platinum compounds and anthracyclines, the same was observed for inhibitors of MK2 [59]. Inhibition of Chk1 and Wee1 in combination was particularly effective [1, 45, 46] but faces the risk of unacceptable toxicities, which may be ameliorated by pre-treatment with Mdm2 inhibitors. Furthermore, substances with predominant toxicity to cells in mitosis are no longer limited to taxanes. Rather, tubulin stabilizers like epothilones as well as signaling inhibitors that preclude a smooth transition through mitosis, e. g. inhibitors of aurora or polo-like kinases [60], also represent suitable candidates for combination with Mdm2 antagonists, to limit their toxicities to normal cells. All these approaches would take advantage of the most commonly encountered genetic difference between human malignancies and non-transformed cells, i. e. a mutation in *TP53*, to tailor therapeutic strategies specifically towards cancer cells and away from other cells in a patient's body. In this way, it is expected that therapies will not only become more tolerable to a patient, but that the maximum doses of tumor-drugs can be augmented to increase therapeutic efficacy.

As a word of caution, the side effects of Mdm2 antagonists need to be considered in these strategies as well. At present, not much is known about how well such antagonists are tolerated, but a dozen phase I studies with Mdm2 antagonists have been registered (clinicaltrials.gov). It is conceivable that Mdm2 inhibition may increase the death of those cells that are particularly sensitive towards p53 (an unwanted on-target effect). A recent study on mice with a global but inducible genetic ablation of Mdm2 revealed that such sensitive tissues not only involve the bone marrow and the gut, but also the kidney [61]. However, an important difference between this model and pharmacological antagonists is the transient nature of the latter. While the genetic ablation of Mdm2 is complete and permanent, pharmacological Mdm2-inhibitors can abrogate Mdm2 activity to an extent that can be adapted to the situation, and Mdm2 can quickly revert to normal p53 antagonism after discontinuing drug administration. The impact of Nutlin alone on the survival was only moderate in most cases of a panel of p53-proficient cell lines [62, 63]. Only in cells with high amplifications of the Mdm2 gene, cells appear to become addicted to this oncogene, rendering them exquisitely sensitive to Nutlin [62]. Since such amplifications are not present in normal cells, we expect that toxicities of Mdm2-antagonizing drugs will be manageable. The same is true for other reported effects of Nutlin, such as DNA breakage [64]or endoreduplication [65] in some cell lines. However, careful assessment of ongoing clinical trials involving Mdm2 antagonists will be required. This will then help to avoid unwanted toxicities by adapting the drug doses and schedules, and possibly by chemically modifying the drugs to reduce their impact on p53-sensitive normal tissues. Moreover, the combination of Mdm2 antagonists with Wee1 inhibitors will require evaluation in animal models before being taken to the clinics.

Taken together, our results suggest that p53 is an important determinant of how Wee1 inhibitors can be used in the clinics. On the one hand, p53 activation in tumor cells must be avoided to prevent negative drug interference with Wee1 inhibitors when a tumor carries wild type p53. On the other hand, however, in p53-mutant tumors, the administration of an Mdm2 antagonist appears as a highly promising opportunity to circumvent the toxicities of Wee1 inhibitors and many other drugs that act in a cell cycle specific manner.

## MATERIALS AND METHODS

### Culturing of human cell lines

U2OS (human osteosarcoma) and HCT116 (colorectal carcinoma) cells were cultured in DMEM and McCoy's, respectively,with 10% FCS, 200μM L-glutamine and antibiotics – 50U/ml Penicillin and Streptomycin, and 10μg/ml Ciprofloxacin (Bayer). In addition, medium for U2OS cells contained 20μg/ml Tetracycline. All media and chemicals except Ciprofloxacin were from Invitrogen. MCF10A (non-transformed breast epithelial) cells were cultured in DMEM/F-12 with 5% horse serum (Sigma H1138), 0.5μg/ml hydrocortisone (Sigma H-0888), 0.1μg/ml cholera toxin (Sigma C-8052), 20ng/ml Human EGF(Sigma E-9644), and 1:1000 Insulin (Sigma I-9278).

### Preparation of whole cell lysates

Cells were seeded in 6-well plates (1.6 × 10^5^ cells per well) for the drug treatment. Cell lysates were prepared on ice. The cells were scraped off into the medium and pelleted by centrifugation at 1500xg for 3 min at 4°C, followed by one wash in PBS. The cells were resuspended in 100μl RIPA lysis buffer (1% Triton X, 1% desoxycholate, 0.1% SDS, 150 mMNaCl, 10 mM EDTA, 20 mMTris-HCl pH 7.5, 100.000KIE Aprotinin) freshly supplemented with 2M urea, 1mg/ml leupeptine/aprotinine, 0.1M pepstatin A, 0.1M pefabloc. After 20 min of shaking at 4°C, the lysates were centrifuged at 15,700xg for 10min. Bicinchoninic acid (BCA) assay was used to normalize the concentration of proteins in the supernatant. The samples were then boiled with Laemmli buffer, followed by SDS-PAGE.

### Transfection of human cells

Using lipofectamine 2000, we carried out transient transfection of U2OS cells with siRNA to knock-down p53, and a corresponding control siRNA as a control. Lipofectamine and siRNA were dissolved separately in DMEM only (without FCS, or and antibiotics) and incubated at room temperature (RT) for 5 min. They were then combined and incubated for another 20 min at RT. In one well of a 6-well plate, 280,000 cells were seeded in 1.6 mL DMEM with 10% FCS, and 400 μL of the lipofectamine-siRNA mix was added drop-wise, followed by a 48 hrs incubation.

### Immunoblot analysis

Blots on nitrocellulose or PVDF membranes were stained with the following antibodies. Phosphorylated Ser 139 H2AX (05-636, Millipore), PARP (9542, Cell Signaling Technology), beta-Actin (ab6276-100, Abcam), phospho-H3 Ser 10 (3377, Cell signaling), p53 (sc-126, Santa Cruz Biotech), p21 (OP64, Calbiochem). Secondary antibodies coupled to horseradish peroxidase H3 (ab1791, Abcam) (Jackson Immunoresearch) were used for chemiluminescent detection (Millipore).

### Immunofluorescence analysis

For immunofluorescence microscopy, the automated microscope Pathway 855 (Becton Dickinson, Franklin Lakes, NJ, United States) was used to read fluorescence intensity in 96-well plates. For confocal microscopy, the LSM 510 laser scanning microscope (Carl Zeiss, Germany) was used.

The cells were fixed in 3.7% paraformaldehyde for 20 min, followed by permeabilization with 0.5% Triton-X in PBS for 15 min and blocking for 15 min using blocking solution (3% BSA in PBS). The primary antibody to phospho-H2AX (05-636, Millipore), diluted in blocking solution, was added for 1 h, followed by incubation with a secondary antibody (Alexa-Fluor 546) and Hoechst 33342 (Invitrogen) diluted in blocking solution for 45 min.

For EdU staining, permeabilization was followed by exposure to Click- iTEdU reaction cocktail (C10351, Invitrogen) for 30 min. The cell nuclei were counterstained with Hoechst 33342.

Images were captured and analyzed using the BD Pathway software, wherein the region of interest (ROI), in this case the cell nuclei, were defined by Hoechst stain, and the average intensity of the antibody-coupled fluorescence within each ROI was determined.

### Caspase activity assay

Cells were seeded in 6-well plates (1.6 × 10 (to the power of 5) cells per well) and treated with drugs. 24 hrs post-treatment, the cells were harvested (inclusive of medium) and centrifuged at 1500xg for 5 min at 4°C. The pelleted cells were resuspended in 250μl caspase lysis buffer (1M Tris-HCl, 2mM MgCl_2_, 150mM NaCl, 10mM DTT, Roche complete mini protease-inhibitor mix). They were shock-frozen thrice in liquid nitrogen and centrifuged at 15,000xg for 15 min at 4°C. 40μl of lysate per well in a 96-well plate was distributed in triplicates. 10μl of Ac-DEVD-AMC substrate (working concentration 25μM) (ALX-260-031 Enzo) was added to each sample. Caspase activity was measured using a fluorometer (Synergy MX 267137) at excitation wavelength 380nm and emission wavelength 460nm every 10 min for 4 hrs at 37°C.

### Cell cycle analysis by flow cytometry

Cells were seeded in 6-well plates and treated with the drugs. After fixation in ethanol, the cells were washed in wash solution (0.05% Triton-X in PBS), followed by incubation in staining solution (2% FCS, 0.2% Triton-X in PBS) with phospho-H3 antibody (3377, Cell signaling) for 2 hrs and then with secondary antibody (coupled to Alexa-Fluor 488) for one hour. Subsequently, the cells were resuspended in 0.5 mg/ml RNAse A solution andincubated for 30 min at 37°C. Directly before measurement, propidium iodide (final concentration: 30 μg/ml) was added. Samples were measured using the flow cytometer Guava PCA-96 Base System (Millipore).

### Cell proliferation assay

Cells were seeded in 96-well plates or 24-well plates, treated after 18-24 hrs, and the confluency of the cells was measured using a Celigo cell cytometer (Cyntellect; labeled as Day0). After 24 hrs, the medium was replaced with fresh media; the confluency was determined again (Day1); subsequent measurements were made every 24 hrs and media was changed every 48 hrs.

### Cell viability assay

Cells were seeded in 96-well plates with white walls and bottom and treated after 18-24 hrs. Cells treated with DMSO in a concentration which responds to the highest concentration of the drugs added were used as a control. Remaining wells without cells were filled with medium in order to obtain a value for background luminescence. Each experiment was incubated for 72 hrs. For measuring the luminescence, the CellTiter-Glo®Luminescence Cell Viability Assay (Promega) was used. CellTiter-Glo®Reagent was added in a 1:1 ratio to the cell culture medium in a well. The plate was placed on an orbital shaker for 10 min for induction of cell lysis. Subsequently, the luciferase signal was measured on a LuminometerDLReady™Centro LB 960 reader.

### Statistical analysis

Statistical significance was determined using the unpaired, two-tailed Student's T-test. Significance was assumed for p-values below 0.05. Asterisks in figures indicate resulting p-values as follows: * p < 0.05, ** p < 0.01, *** p < 0.001. *n.s.* = not significant. *n* in figure legends indicates the number of independent experiments.

## SUPPLEMENTARY MATERIAL FIGURES


