# Supplementary material for: Mdm2 inhibition confers protection of p53-proficient cells from the cytotoxic effects of Wee1 inhibitors
Source: Oncotarget. 2015 Sep 29;6(32):32339–52. doi: 10.18632/oncotarget.5891 (PMC4741697; doi:10.18632/oncotarget.5891)
Supplement: Supplementary file 1 [file oncotarget-06-32339-s001.pdf]

Mdm2 inhibition confers protection of p53-proficient cells from the cytotoxic effects of Wee1 inhibitors

Supplementary Material

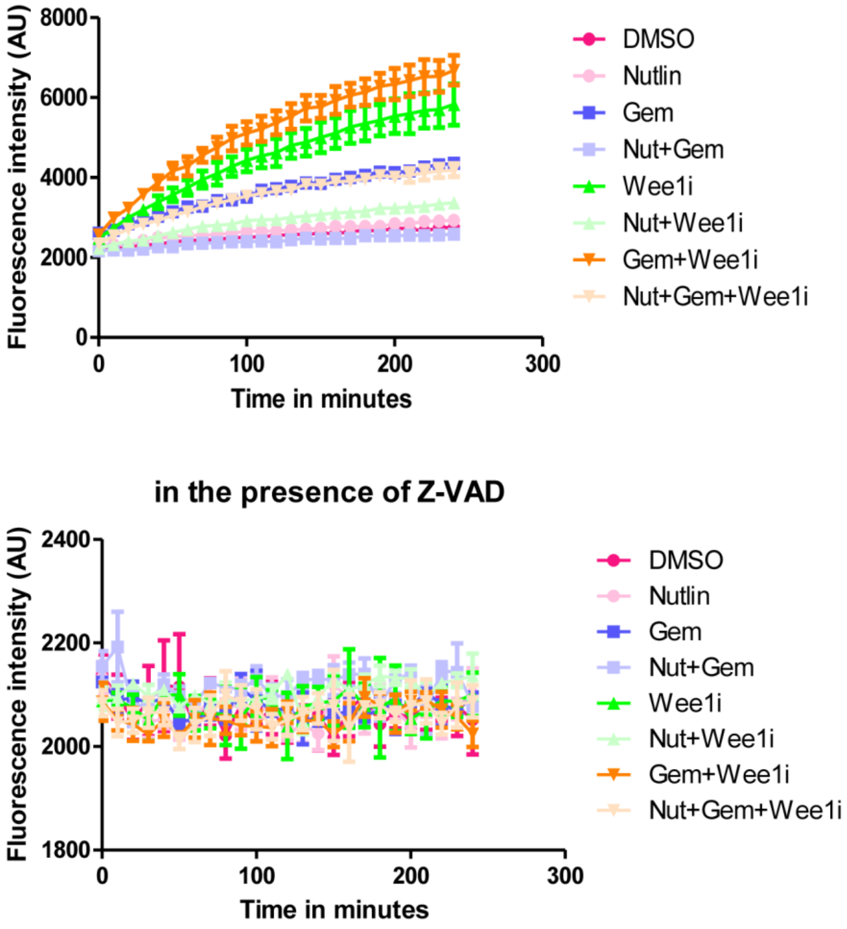

Supplemental Figure 1. Caspase activity upon drug treatment

U2OS cells were treated with 8µM Nutlin for 24 hrs, followed by treatment with 1µM Wee1 inhibitor, 300nM gemcitabine, 8µM Nutlin in the absence and presence of 50µM ZVAD-FMK for another 24 hrs. Cells were harvested and lysed for caspase activity assay. Fluorescence intensity measurements were obtained for each treatment.

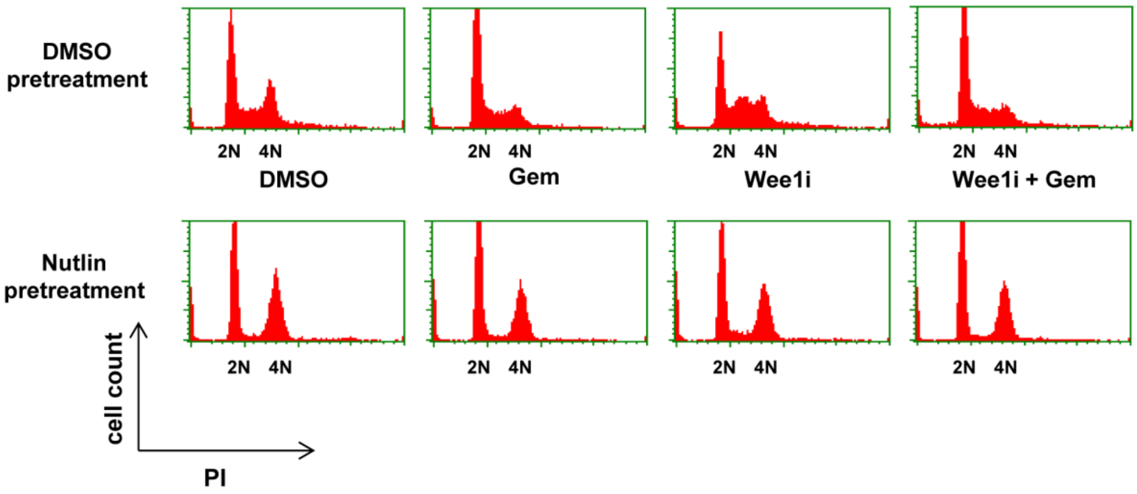

Supplemental Figure 2. Nutlin reduces the amount of cells in S phase

U2OS cells were treated as in Fig. 4A, followed by flow cytometry analysis of the DNA content. Histograms depict the relative number of cells found within a small window of DNA content, as determined by propidium iodide stain. The relative amount of cells with a DNA content between the G1 and G2/M peaks is reduced by Nutlin pretreatment, regardless of subsequent treatment with gemcitabine and/or Wee1i, arguing that Nutlin pretreatment largely precludes entry into S phase.
